# Fractal Analysis of Brain Blood Oxygenation Level Dependent (BOLD) Signals from Children with Mild Traumatic Brain Injury (mTBI)

**DOI:** 10.1371/journal.pone.0169647

**Published:** 2017-01-10

**Authors:** Olga Dona, Michael D. Noseworthy, Carol DeMatteo, John F. Connolly

**Affiliations:** 1 McMaster School of Biomedical Engineering, McMaster University, Hamilton, Ontario, Canada; 2 Imaging Research Centre, St. Joseph’s Healthcare, Hamilton, Ontario, Canada; 3 Department of Electrical and Computer Engineering, McMaster University, Hamilton, Ontario, Canada; 4 Department of Radiology, McMaster University, Hamilton, Ontario, Canada; 5 School of Rehabilitation Medicine, McMaster University, Hamilton, Ontario, Canada; 6 Department of Linguistics, McMaster University, Hamilton, Ontario, Canada; Roskamp Institute, UNITED STATES

## Abstract

**Background:**

Conventional imaging techniques are unable to detect abnormalities in the brain following mild traumatic brain injury (mTBI). Yet patients with mTBI typically show delayed response on neuropsychological evaluation. Because fractal geometry represents complexity, we explored its utility in measuring temporal fluctuations of brain resting state blood oxygen level dependent (rs-BOLD) signal. We hypothesized that there could be a detectable difference in rs-BOLD signal complexity between healthy subjects and mTBI patients based on previous studies that associated reduction in signal complexity with disease.

**Methods:**

Fifteen subjects (13.4 ± 2.3 y/o) and 56 age-matched (13.5 ± 2.34 y/o) healthy controls were scanned using a GE Discovery MR750 3T MRI and 32-channel RF-coil. Axial FSPGR-3D images were used to prescribe rs-BOLD (TE/TR = 35/2000ms), acquired over 6 minutes. Motion correction was performed and anatomical and functional images were aligned and spatially warped to the N27 standard atlas. Fractal analysis, performed on grey matter, was done by estimating the Hurst exponent using de-trended fluctuation analysis and signal summation conversion methods.

**Results and Conclusions:**

Voxel-wise fractal dimension (FD) was calculated for every subject in the control group to generate mean and standard deviation maps for regional Z-score analysis. Voxel-wise validation of FD normality across controls was confirmed, and non-Gaussian voxels (3.05% over the brain) were eliminated from subsequent analysis. For each mTBI patient, regions where Z-score values were at least 2 standard deviations away from the mean (i.e. where |*Z*| > 2.0) were identified. In individual patients the frequently affected regions were amygdala (p = 0.02), vermis(p = 0.03), caudate head (p = 0.04), hippocampus(p = 0.03), and hypothalamus(p = 0.04), all previously reported as dysfunctional after mTBI, but based on group analysis. It is well known that the brain is best modeled as a complex system. Therefore a measure of complexity using rs-BOLD signal FD could provide an additional method to grade and monitor mTBI. Furthermore, this approach can be personalized thus providing unique patient specific assessment.

## Introduction

Mild traumatic brain injury (mTBI), commonly referred to as concussion, is a significant medical condition with serious implications for those affected, particularly where the extension of the injury is not easily assessed or even detected. It represents 75% of all head injuries [1] and disproportionally affects young men and athletes. Furthermore, the elderly are also a significant group because of increased incidence of falls and subsequent head trauma [2, 3].

All mTBIs are neurological injuries, which occur from rapid accelerative and/or decelerative linear and rotational forces applied to the head. These result in rapid velocity changes leading to the brain hitting the inside surface of the skull on the side nearest the origin of the force. Subsequently, milliseconds later, the brain also collides with the opposite side of the skull (contra-coup injury). In addition to direct translational forces there are also rotational and shearing forces making mTBI a highly complex injury. The occurrence of these forces does not necessarily come from direct impact. The subsequent pathology is thought to be the result of a primary mechanism (i.e. shearing and compression) occurring at the time of injury and from a secondary mechanism involving edema and hypoxia, which occur in a time window of hours to days after the incident [4]. Post-concussive symptoms are generally grouped into three categories: cognitive, physical and behavioral anomalies. Symptoms include headache, difficulty concentrating, sleep impairment, memory deficit, depression and anxiety [5, 6]. These clinical symptoms are persistent in 10% of cases, lasting for months to years after the injury [7].

mTBI can be defined as a range of microstructural injuries coupled with functional disturbances, rather than major structural injury, and thus have minimal detectable anatomic pathology [8]. Consequently, conventional magnetic resonance imaging (MRI) and computed tomography (CT) techniques are ineffective for mTBI assessment, whether the patient has persistent post-concussive symptoms or not. Therefore, the main objective of our work was to focus on developing a new approach to assess brain functional disturbances. To do this we exploited the functional capabilities of the resting state blood level oxygen dependent (BOLD) signal obtained with magnetic resonance imaging (MRI).

The BOLD effect results from differences in magnetic properties between oxyhemoglobin and deoxyhemoglobin in blood vessels. The magnetic susceptibility difference between these two results in a T2*-weighted signal change. Under an external stimuli, neuronal activity increases, creating a demand for oxygen delivery and therefore increasing the BOLD signal. Furthermore, in the absence of a specific task or stimuli, BOLD signals show fluctuations as a result of complex brain interactive and connective function and this is known as resting state BOLD (rs-BOLD). The rs-BOLD signal is a complex signal, where the effects of cerebral blood oxygenation and cerebral blood flow and volume are convoluted. The rs-BOLD signal shows spontaneous low frequency fluctuations [9], that exhibit inverse power-law scaling in the frequency domain [10]. It has been suggested, that these fluctuations and the inverse power-law behavior they follow originate from physiological functions as well as from instrument noise added during fMRI acquisition [10–14]. The inverse power-law scaling is a fundamental characteristic of signals or structures considered as fractals. Fractals are infinitely complex patterns that are self-similar across different scales and their estimated dimension is a measure of the complexity of the system [15]. Complexity of the BOLD signal has been previously used as a descriptor of the neural activity based on hemodynamics and metabolic response [12, 16]. The brain, when healthy, is best described as a complex system and thus could display regional changes in the fractal dimension (FD) of the rs-BOLD signal as a result of a mTBI. A direct injury to the white matter tracts and demyelination due to chronic inflammation could affect structural connectivity among certain regions and inflammation around deep brain structures could also affect neuronal activity [17]. We hypothesized that FD could be a measure of brain local neural activity and therefore be a metric for identifying subtle functional changes in mTBI patients that are not appreciable at the anatomical level.

## Materials and Methods

### Patients and controls

Fifteen subjects (13.4 ± 2.3 y/o) with a diagnosis of concussion (post-concussion symptom scale, PCSS = 29.9 ± 23.8) and 56 age-matched (13.7 ± 7.8 y/o) healthy controls were scanned at rest with eyes open. For the mTBI patients the average time between MRI scanning and the date of injury was 33.0 ± 43.8 days. The study was approved by our Institutional Research Ethics Board, (Hamilton Integrated Reaseach Ethic Board (HIREB)) and all patients gave written informed consent. As these patients were paediatric, parental assent was also approved in writing. The study was conducted according to the principles expressed in the Declaration of Helsinki.

### Data acquisition and pre-processing

Patients were scanned using a GE MR750 Discovery 3T MRI scanner, while all control data were scanned using a similar GE Healthcare 3T MR scanner. Both systems used a 32-channel RF receiver coil (General Electric Healthcare, Milwaukee, WI). Healthy control data were obtained from the NIH database (ABIDE-Michigan_S1) [18]. Control data were acquired in a similar MRI scanner, using the same pulse sequence and parameters as in the patients data.

Following a routine 3-plane localizer and calibration scan for parallel imaging, a 3D inversion recovery-prepped T1-weighted anatomical data set was acquired (fSPGR, axial acquisition, TE/TR/flip angle = 4.25/11.36/12°, 256x256 matrix with 1mm slice thickness with 25.6cm FOV, 1mm isotropic acquisition). Resting state functional BOLD data was acquired using an echo planar imaging (EPI) sequence with FOV = 22cm, image matrix = 64x64; flip angle = 90°; echo time (TE) = 35ms; repetition time (TR) = 2000ms (i.e, 0.5Hz temporal sampling frequency); slice thickness of 3mm; and 180 temporal points. At the beginning of every scan, 4 additional data points were acquired but automatically discarded (allowing the system to reach steady state), making the final scan time 6 minutes and 8 seconds.

Motion correction was performed on resting state data, using a 6 point affine transformation, with the AFNI tool 3DVolreg. Images were spatially registered to the first volume of the rs-BOLD data. Then anatomical and motion corrected rs-BOLD data were aligned and spatially warped using a 12-point affine transformation to the TT_N27 atlas using AFNI [19]. Because functional information is assumed to be processed predominantly in gray matter a binary mask was created from the TT_N27 atlas and multiplied through all functional rs-BOLD volumes.

### Fractal analysis

Fractal analysis, performed over the mask on a voxel-wise basis, was done by calculating the Hurst exponent according to the procedure described by Eke et al. [13], using Matlab (v.8.3.0, The Mathworks, Natick MA). According to the rs-BOLD acquisition parameters, the time signal was sampled at 0.5 Hz for 360 seconds (180 time points x TR). Fractality or self-similarity of the time signal was assessed using a power spectral density (PSD) analysis. Low frequency fluctuation (LFF) of the BOLD signal follows an inverse power law scaling according to Eq (1) [14]:

$$\begin{array}{c} |A{\left(f\right)}^{2}|\propto cf^{-\beta }, \end{array}$$ (1)

Where *A* is the amplitude of the discrete Fourier transform (DFT) at frequency *f*; *β* is the spectral index and *c* is a constant. Following the dichotomous model proposed by Mandelbrot and Van Ness [20] the signal can be classified as fractional Brownian motion (fBm) for *β* > 1 and fractional Gaussian noise (fGn) for *β* < 1, with the Hurst exponent (H) calculated as *H* = (*β* − 1)/2 and *H* = (*β* + 1)/2 respectively for classification purposes. H is a measure of the correlation or anti-correlation of the signal. When H is close to 1.0 this indicates high correlation while H closer to 0 indicates anti-correlation. The long-memory dependence characterized by the Hurst exponent is a global characteristic while the fractal dimension is a local property. Local properties are reflected in the global characteristics, for self-affine processes in an n-dimensional space which result in the relationship *FD* + *H* = *n* + 1 where *n* = 1 for a time domain signal.

**Fig 1:**
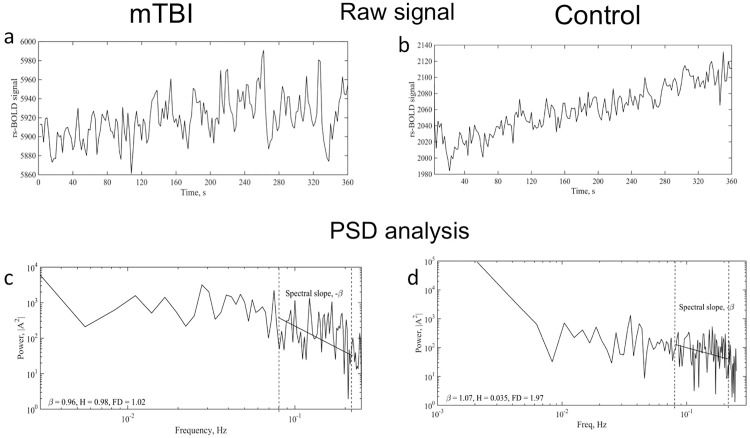
Raw signal and power spectrum analysis. (a) Sample raw rs-BOLD signal from grey matter of an mTBI patient(a) and a healthy control brain(b). These time courses were specifically from a 4x4x3mm voxel in the right hippocampus, located at 24.0[L], 5.0[P], 15.0[S] mm in the N27 atlas. (c,d) Power spectrum, from the same voxel from Fig 1a and Fig 1b respectively, showing power-law decay on a log-log scale. A frequency range of 0.08–0.16 Hz was fit because of the consistency in power law scaling behavior (between and within subjects) of this spectral region.

Following temporal co-registration, the raw BOLD signal Fig 1a was normalized (voxel-wise), end matched and bridge de-trended based on the procedure described by Eke et al. [14]. The log-log representation of the power spectrum Fig 1b contained multiple characteristic regions, which could be a sign of multi-modality [12]. However, for the purpose of our study, we selected a frequency range from 0.08–0.16 Hz where power-law scaling behavior was consistently observed across all voxels and subjects. We followed recommendations from Herman et al. [12] about excluding low frequency regions of below 0.02 Hz due to the presence of MRI system noise in that region [10]. The peak at 0.1 Hz caused by synchronized vasomotion was not excluded as a previous study suggested in only appears in about 2% of the acquisitions [12]. The same study reported that arterial blood pressure does not affect the fractal analysis as it is uncorrelated with the rs-BOLD fluctuations.

The final Hurst coefficient was calculated by applying dispersional analysis on the fGn signals; scale windowed variance (SWV) analysis on the fBm signals and signal summation conversion (SSC) methods for the un-classified signals. FD maps were generated using the estimated voxel-wise Hurst exponent for the 15 patients and the 56 controls.

### Statistical analysis

**Fig 2:**
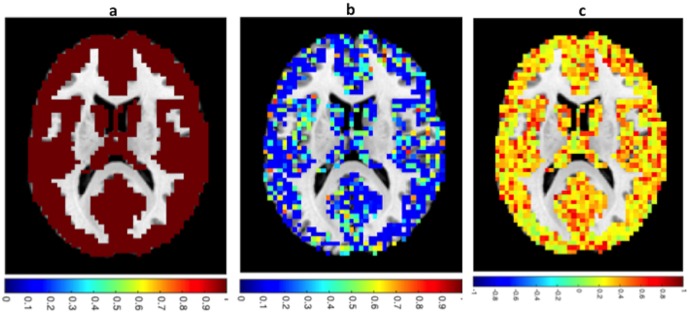
Analysis of normality. (a) Kolmogorov – Smirnov normality test. h = 1 indicates rejection of the null hypothesis at a 5% significance level. (b) Kurtosis map. Voxels where *k* ≠ 3.0 ± 0.5 were removed from analysis. The map was centered at k = 3. (c) Skewness map. Skewness measures asymmetry of the distribution. Positive skew(sk) indicates more data points above the mean while negative skew indicates more data points below the mean. Voxels where *sk* ≠ 0.0 ± 0.5 were removed from the analysis and the sk map was normalized.

A voxel-based Z-scoring methodology was used for statistical analysis. The Z-score is the number of standard deviations (*σ*) a data point is above (*Z* > 0) or below the mean (*Z* < 0). The Z-score of the voxel-wise fractal dimension was calculated as: *Z*_*FD*_ = (*x* − *μ*)/*σ*. Where x is the localized voxel rs-BOLD FD and *μ* and *σ* are the voxel mean and standard deviation of that same voxel from the control group respectively. Prior to application of Z-scoring, voxel-wise validation of normality was performed on the control group data (i.e. voxel-wise skewness and kurtosis was investigated). Failure to satisfy normality indicated voxels that were not classifiable based on this approach. Also, due to inaccurate spatial warping of all control subjects some voxels had the possibility of not existing (i.e. unable to classify) simultaneously over all control subjects. To achieve a statistical power of at least 0.9 only voxels that existed simultaneously in at least 11 subjects were included in the final Z-score maps. Control data was tested for normality using the Kolmogorov-Smirnov test [21] Fig 2a, and kurtosis and skewness calculations Fig 2b and 2c. Based on the Kolmogorov-Smirnov test control data was considered normal within the gray matter mask. However, a more detailed analysis of skeweness and kurtosis revealed voxels that deviated from the univariated normal distribution, which were subsequently removed from the final Z-score map. This approach removed only 3.06% of the voxels in the mean mask for normal controls. Thus the final mask was deemed acceptable for use as a Z-score featurespace.

### Regions of interest(ROIs)

**Fig 3:**
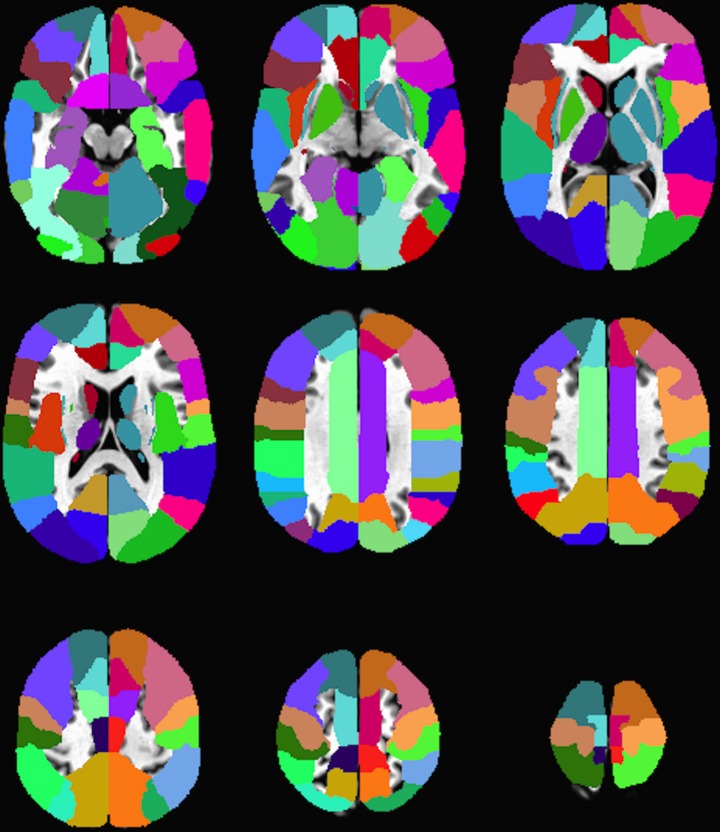
Regions of interest. Montage showing 9 axial slices (taken every 5mm) through the TT_Daemon human brain atlas [22] with a selection of the 240 colour-coded brain structures identified.

The Z-score maps from patients were co-registered to the TT_Daemon [22] human brain atlas and the mean Z-score was calculated for each of the 240 regions included in the atlas Fig 3.

## Results

### Fractal dimension maps

**Fig 4:**
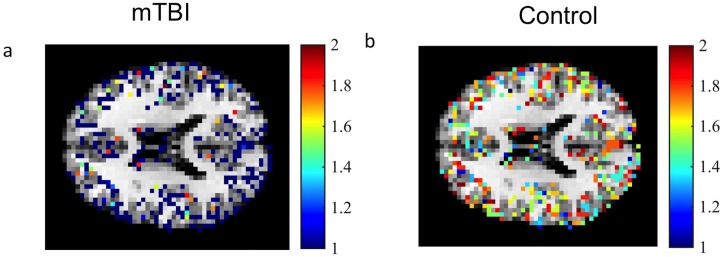
FD map. FD map over a gray matter mask for an mTBI patient(a) and a healthy control(b). FD values closer to 2 show increased signal complexity while FD values closer to 1 show decreased signal complexity in that region.

Voxel-wise FD values were calculated for each of the 15 patients and 56 controls from the gray matter mask Fig 4. The mean gray matter FD for TBI patients was 1.58 ± 0.03 while the mean gray matter FD for controls was 1.61 ± 0.01. Using an unpaired t-test with unequal sample sizes, overall brain gray matter FD in patients was significantly lower compared to control (*p* < 0.05).

### Z-score and ROI analysis

**Fig 5:**
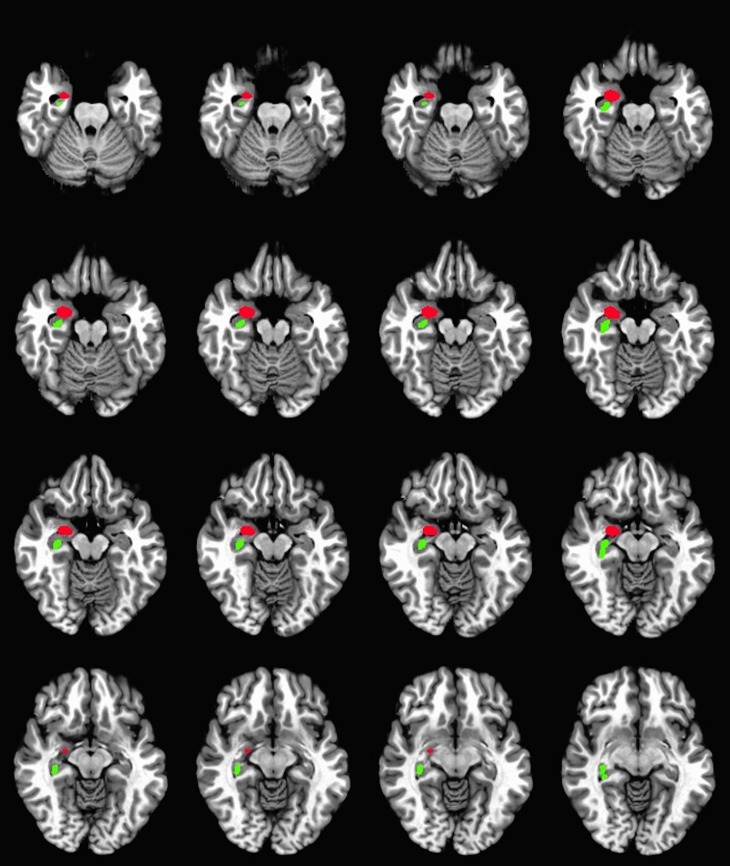
ROIs with |*Z*| > 2.0. Z-score map over grey matter mask was used to calculate the regions that significantly deviated (p = 0.01) from the mean FD. This particular patient showed significant FD decreased in the right hippocampus (red) and the right amygdala (green).

**Fig 6:**
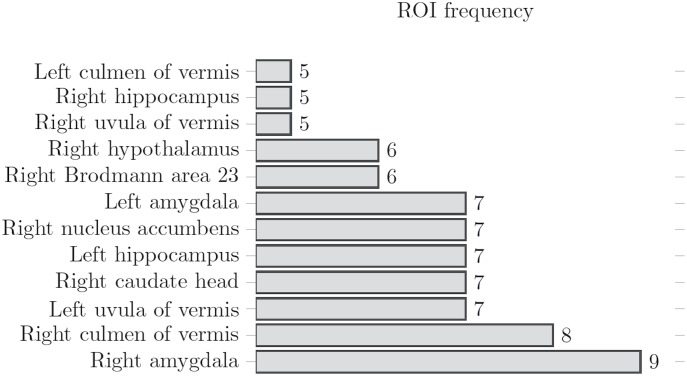
ROI frequency. Bar graph showing ROI frequency in mTBI. Regions where FD decreases significantly. i.e. 9 out of 15 patients showed decreased FD in the right amygdala while 5 out of 15 showed decreased FD in the right hippocampus.

Voxel-wise Z-score maps were calculated for every patient Fig 5 on the 240 defined regions. Ten regions that deviated the most from the control group mean were extracted for each subject and the 11 regions with higher frequency of abnormal fractal behavior were selected as the final regions of interest Fig 6. Table 1 shows mean Z-score, standard deviation and p values calculated for those regions of interest that deviated greatest from control mean values.

**Table 1:** Mean Z-score, standard deviation and p-values for ROI FD values that deviated greatest from healthy controls.

ROIs	*μ*	*σ*	p
Right Amygdala	−2.28	0.90	0.02
Right Culmen of Vermis	−2.40	1.07	0.02
Left Uvula of Vermis	−2.14	1.09	0.03
Right Caudate Head	−2.06	0.70	0.04
Left Hippocampus	−2.28	0.65	0.02
Right Nucleus Accumbens	−1.99	1.11	0.05
Left Amygdala	−2.06	0.68	0.05
Right Brodmann area 23	−2.06	0.52	0.05
Right Hypothalamus	−2.01	0.79	0.04
Right Uvula of Vermis	−1.73	1.24	0.08
Right Hippocampus	−2.04	0.85	0.04
Mean_Z (whole brain GM)	−2.85	0.38	0.004

The PCCS score was collected for every mTBI patient. This is a self-reported questionnaire where patients report, using a scale from 0-6 (no symptoms to severe), symptoms as headache, nausea, vomiting, balance problems, dizziness, fatigue, trouble falling to sleep, excessive sleep, loss of sleep, drowsiness, light sensitivity, noise sensitivity, irritability, sadness, nervousness, more emotional, numbness, feeling slow, feeling foggy, difficulty concentrating, difficulty remembering and visual problems.

Z-scores were calculated for all the regions of interest and correlated with the PCSS score, using the Pearson Product Moment Correlation or PPMC (Table 2), to show whether there is any relationship between the two variables. For the purpose of this study the strength of the correlation was classified in low, moderate and high, following the criteria proposed on Table 3. The p-values reported tested the hypothesis of no correlation against the alternative that there is nonzero correlation. A smaller p-value indicates greater significant difference from zero.

**Table 2:** Pearson correlation coefficients and p-values of PCSS compared against regional rs-BOLD Z-score.

Pearson correlation coefficient /ROIs	r	p
Right Amygdala	0.42	0.14
Right Culmen of Vermis	0.46	0.10
Left Uvula of Vermis	0.26	0.37
Right Caudate Head	0.41	0.15
Left Hippocampus	0.39	0.17
Right Nucleus Accumbens	0.31	0.29
Left Amygdala	0.11	0.70
Right Brodmann area 23	0.29	0.32
Right Hypothalamus	0.10	0.74
Right Uvula of Vermis	0.45	0.11
Right Hippocampus	0.25	0.40
Mean_Z (whole brain GM)	0.54	0.05

**Table 3:** Qualitative criteria used to determine strength of the correlation between FD and PCSS.

Strength of Correlation	Pearson correlation coefficient, r
Low	0.1-0.3
Moderate	0.3-0.5
High	0.5-1.0

## Discussion

Deoxyhemoglobin, because of its paramagnetic properties compared to diamagnetic oxyhemoglobin, has a shorter T2* relaxation time and hence reduced MR signal. When an area of brain is activated, the local neuronal oxygen consumption increases leading to a requirement for increased oxygen delivery (i.e. oxyhemoglobin). This is accomplished by a disproportionate increase in blood flow and volume, in other words more oxygen is delivered during activation than what is metabolically required. This leads to a downstream decrease in deoxyhemoglobin and corresponding elevation in T2* and MR signal. Because the signal change is due to an increased ratio of oxy to deoxyhemoglobin it is called the blood oxygen level dependent BOLD signal. During a specific task BOLD increases [23]. However, in the absence of a task, BOLD signal fluctuates as a result of complex brain interactive and connective function. This is referred to as rs-BOLD.

Based on time-domain correlation analysis, rs-BOLD has shown how parts of the brain are regionally in temporal synchrony. These so called resting state networks are robust and seen in all healthy brains. A number of networks are easily found using region-of-interest (ROI) based localization. For example, the default mode network (DMN), the most dominant resting state network in the brain, is assessed through probing time domain correlation between the posterior cingulate and all other areas of the brain [24]. The problem with typical rs-BOLD analysis is that concurrent assessment of a large number of networks, especially the more subtle ones, requires a large number of concatenated 4D brain data sets and subsequent assessment via probabilistic independent component analysis (PICA). Such an approach is thus only for group-based analysis and single subject evaluation is impossible. An alternative single-subject approach, using rs-BOLD, is using model-free complexity analysis. Measurement of the rs-BOLD fractal dimension is the most frequently avenue for complexity analysis. This method can be used for single subject analysis and is temporally stable within, and between, healthy subjects [25].

Previous analysis of rs-BOLD signals using complexity analysis, based on fractals, has been done to assess early onset Alzheimer’s disease (AD). In this work Warsi et al. [26] studied the correlation of rs-BOLD fractal dimension and in vivo proton magnetic resonance spectroscopy (^1^H-MRS) in the left putamen of AD patients and normal controls. It was shown that decreased FD was consistent with AD severity, as measured with known biomarkers N-acetyl aspartate (NAA) (r = 0.44, p = 0.015) and myoinositol (myoI) (r = -0.45, p = 0.012). Additionally, a study by Weber et al. [27] showed how complexity of the rs-BOLD signal decreased as ethanol levels in the brain increased. This was seen particularly in the right basal ganglia at 60 and 90 minutes after ethanol consumption. Furthermore, the rs-BOLD signal complexity returned as the brain ethanol became metabolized. Ethanol directly affects function of the brain GABA-A receptors [28] decreasing brain connectivity. As ethanol was cleared in the brain, brain connectivity increased and fractal dimension increased. The conclusion from this work was that the temporal complexity of brain resting state was diminished with increased levels of brain alcohol. In other words, the ability of the brain to process information was reduced in an intoxicated state and this effect was observed in the fractal dimension of the BOLD signal.

Complexity of brain signals, characterized by the fractal dimension, has also been investigated in resting-state electroencephalography (rs-EEG). A recent study by Smits et al. [29] showed calculations of FD in resting state EEG recordings from 67 AD patients and 41 healthy controls. They found the FD and signal complexity decreased with age in normal controls and that it further decreased in AD patients, especially in temporal-occipital regions [29]. This study supports the concept that brain signal FD correlates with changes in brain connectivity and complexity.

Based on previous studies [12, 26, 27], it can be inferred that the FD of rs-BOLD represents brain temporal complexity at rest. Ideally, a healthy brain could be associated with more complex signals, due higher multi-level and multi-time connectivity within different brain regions. Furthermore, FD could be an indicator of the brain’s ability to perform real-time adaptation and processing of the multitude of external stimuli that subsequently lead to the continuous driving of brain metabolic fluctuation. Low FD characterizes less complex signals, which has been associated with pathologies of the brain [26–28, 30]. Therefore, a decrease in signal complexity could be associated with lack of adaptability and decreased brain connectivity.

The fractal dimension maps produced for mTBI and control subjects showed that overall, grey matter rs-BOLD FD in mTBI patients decreased compared to controls. This indicates reduction in temporal complexity of the rs-BOLD leading us to our hypothesis that patients with mTBI experience a decrease in brain connectivity and that this could be observed with the FD approach. Z-score and subsequent regional ROI analysis revealed a group of brain regions where FD values were observed to deviate the greatest from mean values, as calculated from a population of healthy controls. On average these regions had Z-scores values of -2.09 ± 0.18, or 2.09*σ* below the mean. Table 1 shows 11 brain regions where FD significantly decreased for mTBI patients.

In nine out of fifteen mTBI patients, the right amygdala was among the ten regions with lower Z-score values, however the right culmen of vermis reported the lowest values among the all the studied ROIs. The amygdala is a brain structure known to be highly involved in the processing of emotions. Animal studies have shown decreased excitability, decreased activation and inflammation in the amygdala after mTBI [31, 32]. The reported effects of mTBI in the amygdala also validate neuropsychological symptoms commonly reported by TBI patients. Our study showed decreased FD in the amygdala (Z-score = -2.28 ± 0.90), which is consistent with decreased neural activity in the region.

The culmen and the uvula of vermis, both cerebellar structures had decreased FD values (Z-score = -2.40 ± 1.07 and Z-score = -1.93 ± 1.16 respectively), supporting previous studies that suggested a link between mTBI and cerebellar dysfunction. A recent diffusion tensor imaging (DTI) study of mTBI patients showed decreased fractional anisotropy (FA) in the vermis compared to normal controls [33]. Lower FA has been implicated in reduction of myelin integrity, which for mTBI likely would be due to shearing forces causing micro tears in the axons. [33]. They suggested that damage on these cerebellar regions could be associated with a dysfunction in primitive fear conditioning circuits [34]. Additionally, a longitudinal study based on brain volume changes, showed a significant decrease in cerebellar volume of mTBI patients [35].

The caudate nucleus and nucleus accumbens are regions of the basal ganglia. They have been implicated with voluntary movement, learning, memory, sleep, and social behavior and cognitive processing of aversion, motivation, pleasure, reward and reinforcement learning respectively [36, 37]. We found decreased FD for these two regions and Z-scores were -2.06 ± 0.7 and -1.99 ± 1.11 respectively. Decreased signal complexity in these regions agrees with neuropsychological symptoms commonly reported by mTBI patients. A previous study correlated iron deposition in these regions, through susceptibility weighted imaging (SWI), to cognitive impairment in mTBI patients. A significant increase in caudate nucleus iron deposition has been found to be positively correlated with the mini-mental state examination [38].

There is increasing evidence that cognitive and memory dysfunction of mTBI patients is related to neuro-physiological changes that occur in the hippocampus. Recent studies have shown changes in important neurotransmitters such as glutamate and *γ*-aminobutyric acid (GABA) in the hippocampus following mTBI [39, 40]. We found that FD decreased for mTBI patients when compared with the uninjured control group in both these areas. The mean Z-score value for hippocampus was -2.16 ± 0.75. Decreased signal complexity in the hippocampus after mTBI is consistent with changes in neuronal firing patterns reported by Witgen et al. [41].

ROI-based Z-scores were correlated with PCSS scores using the Pearson Product Moment Correlation (PPMC). The correlation coefficients (r) paired with the respective p-values (Table 2) showed high correlation for GM; moderate correlation for the right nucleus accumbens, right uvula of vermis, left hippocampus, right caudate head, right culmen of vermis and right amygdala; and low correlation for left uvula of vermis, left amygdala, right Brodmann area 23, right hypothalamus and right hippocampus. Negative correlation was expected because we hypothesized that as symptoms worsen the FD should decrease. However, all ROIs and GM showed positive correlation Table 2 with PCSS. This implies that as symptoms worsen the FD Z-score increases (absolute value of Z-score decreases). The absence of significant correlation with the PCSS score was expected because this metric does not characterize symptoms associated with unique brain regions. Still, PCSS is the most common test used clinically to characterize mTBI. This study, highlights the issues related with the use of a self reported metric while trying to characterize a complex phenomenon.

The effectiveness of PCSS in the assessment of cerebral concussion remains unclear given that such symptoms are non-specific. A study by Iverson et al. indicated that non-concussed normal controls have reported identical symptom scores than those used on the PCSS score [42]. Therefore, using PCSS as a metric to establish correlation with mTBI FD data may be considered unsatisfactory. Neuropsychological tests specifically designed to measure a psychological function, related to a particular brain structure or pathway would be of greater interest for future studies.

The main limitation of this study arises from our relatively low sampling frequency. The effectiveness of the fractal analysis is based on the ability of the signal to capture the true dynamics of the processes being studied. Ideally the sampling frequency should be one order of magnitude higher than the highest frequency of the hemodynamic response to neuronal activation. The BOLD responses are delayed by 1–2 s and have a temporal width on the order of 4–6 s [43], therefore we need to be able to sample the signal at 0.125 Hz (ideally 1.25 Hz). In order to acquire the rs-BOLD signal for the entire brain in a reasonable time for the patients we were only able to sample at a frequency of 0.5 Hz which significantly limited the scope of our study. New techniques such as multi-band EPI would be ideal to overcome this issue as they are able to achieve full brain sampling rates up to 2.5 Hz [44].

## Conclusions

This study shows how rs-BOLD fractal dimension appears to provide additional patient-specific brain focal information that can be used to assess and possibly monitor mTBI patients. Traditional functional imaging approaches, based on linear models, are able to show differences between normal and mTBI patients, but only based on group-based statistics. It is well known that the brain is best modeled as a complex system [45] and therefore a measure of complexity using FD could provide a method to approach the mTBI problem. In this study, we were able to find regions in the brain that despite not showing any abnormality in an anatomical scan, reported decreased signal complexity using FD methodology. These regions have been previously reported as dysfunctional for mTBI patients. The method we have proposed is able to provide additional information of mTBI in a non-invasive and fast manner and could hopefully help in the design of future treatment plans.
